# Establishing content validity of the Dimensional Anhedonia Rating Scale

**DOI:** 10.1186/s41687-025-00860-x

**Published:** 2025-03-11

**Authors:** Stephanie Bean, Rahul Dhanda, Christina A. Graham, Deborah Hoffman, Mariam Rodriguez-Lee, Adrian Ionescu, Stella Karantzoulis, Sidney H. Kennedy, Sakina J. Rizvi

**Affiliations:** 1 Patient Centered Solutions, IQVIA, 600 Lexington Avenue, New York, NY 10022 USA; 2 Neurocrine Biosciences, Inc., 12780 El Camino Real, San Diego, CA 92130 USA; 3 Takeda Pharmaceutical Company, 650 E Kendall St, Cambridge, MA 02142 USA; 4 Department of Psychiatry, University of Toronto, 27 King’s College Circle, Toronto,, ON M5S Canada; 5 ASR Suicide and Depression Studies Program, St Michael’s Hospital, 30 Bond Street, Toronto, Canada

**Keywords:** Anhedonia, Major depressive disorder (MDD), Dimensional Anhedonia Rating Scale (DARS), Qualitative interviews, Concept elicitation (CE), Cognitive debriefing (CD), Conceptual model, Content validity

## Abstract

**Background:**

This study was designed to evaluate content validity of the Dimensional Anhedonia Rating Scale (DARS), a patient-reported outcome measure, in adults with anhedonia in the context of major depressive disorder (MDD). To accomplish this, a conceptual model including the symptoms and impacts of anhedonia in the context of MDD was developed and refined through a targeted literature review, clinician interviews (N = 6), and participant interviews (N = 20).

**Results:**

Using the final conceptual model, an item mapping exercise was conducted for the DARS, demonstrating that it provided suitable concept coverage in this population. Cognitive debriefing of the DARS with participants demonstrated that it was generally well understood and clear.

**Conclusions:**

Overall, the study established that the DARS demonstrates content validity in adults with anhedonia in the context of MDD. Other measurement properties of the DARS will be evaluated in planned psychometric analyses.

**Supplementary information:**

The online version contains supplementary material available at 10.1186/s41687-025-00860-x.

## Background

Major depressive disorder (MDD) is estimated to be the leading cause of disease burden worldwide by the end of 2030 [1]. In the USA, the number of adults with MDD increased from 13.8 million to 17.5 million between 2005 and 2018, with a rise in prevalence from 6.4% to 7.1% [2, 3].

Anhedonia, defined as loss of interest or pleasure in activities, is a core symptom of MDD and reported in nearly 75% of patients [4–6]. Anhedonia has been associated with impairments in the reward-processing system in the brain as well as social withdrawal, increased risk of suicide, and poor treatment outcomes with antidepressants [7–12]. Anhedonia has also been shown to impact day-to-day functioning and quality of life of patients with MDD [8, 11, 13–15].

To support development of pharmacotherapies for anhedonia in the context of MDD, the use of suitable patient-reported outcome (PRO) instruments to evaluate efficacy is crucial. PRO instruments are often used to assess anhedonia from the patient perspective and the following instruments have been used in research studies: Snaith-Hamilton Pleasure Scale (SHAPS), Fawcett-Clark Pleasure Capacity Scale, Revised Chapman Physical Anhedonia Scale (CPAS), and Chapman Social Anhedonia Scale (CSAS) [16–19]. Although these scales are widely used in clinical research, and the SHAPS is considered the current gold standard to assess hedonic experiences, they bear limitations related to generalizability, cultural bias, and specificity [18, 20]. To address the limitations of existing PRO instruments for anhedonia, the Dimensional Anhedonia Rating Scale (DARS) was developed. This 17-item self-report scale was designed to assess symptoms of anhedonia (rather than impact of anhedonia) through four domains: hobbies, food/drink, social activities, and sensory experience. By using self-reported examples in item responses, the DARS avoids bias related to culture, age, or gender. The DARS has demonstrated high internal consistency with good convergent and divergent validity, and utility over the SHAPS in predicting treatment-resistant depression [20]. The DARS has also shown an ability to measure reward function, specifically across reward domains, including aspects of consummatory pleasure, interest/desire, motivation, and effort, and is reported to be highly consistent with related scales [21–23].

For a PRO to be useful, it needs to address the scope of content that is relevant to its target population. This requires integrating a qualitative methodological approach into the scale development program. The present study aimed to evaluate the content validity of the DARS through a targeted literature review (TLR), clinician interviews, and participant (i.e., adults with anhedonia in the context of MDD) interviews. The study was designed in alignment with the Food and Drug Administration (FDA) PRO Guidance (FDA, 2009) and Good Clinical Practice (gcp.nidatraining.org) [24, 25].

## Methods

### Targeted literature review

A TLR was conducted to identify patient-relevant concepts (i.e., signs [observable manifestations of the condition], symptoms [internal experiences of the condition], and impacts [effects on daily life and day-to-day functioning due to the signs and symptoms of the condition]) of anhedonia in the context of MDD as well as PRO instruments used in this population. The TLR was executed using search terms (Table 1) and eligibility criteria (Table 2) in four databases: ClinicalTrials.gov, PROQOLID and PROLABELS via ePROVIDE, PubMed, and PyscNet.

**Table 1:** Targeted literature review search strategy

Database/search	Search terms and limits	
PubMed	((anhedonia[Title]) AND (depressive disorder[Title/Abstract] OR MDD[Title/Abstract] OR depression[Title/Abstract])) AND (quality of life OR QoL OR patient report*[MeSH Terms])	
	((anhedonia[Title]) AND (depressive disorder[Title/Abstract] OR MDD[Title/Abstract] OR depression[Title/Abstract])) AND (quality of life[Title/Abstract] OR QoL[Title/Abstract] OR HRQoL[Title/Abstract] OR function*[Title/Abstract] OR impact*[Title/Abstract] OR well being[Title/Abstract] OR general health perception*[Title/Abstract] OR health state*[Title/Abstract] OR self report*[Title/Abstract] OR patient report*[Title/Abstract] OR PRO[Title/Abstract] OR PROs[Title/Abstract] OR diary[Title/Abstract] OR diaries[Title/Abstract] OR patient perspective*[Title/Abstract] OR patient centricity[Title/Abstract] OR patient centered[Title/Abstract] OR patient centered[Title/Abstract] OR interview*[Title/Abstract] OR survey*[Title/Abstract] OR conceptual model*[Title/Abstract] OR disease model*[Title/Abstract] OR focus group*[Title/Abstract] OR literature review*[Title/Abstract] OR systematic review*[Title/Abstract] OR longitudinal[Title/Abstract] OR observational[Title/Abstract] OR cohort*[Title/Abstract] OR case series[Title/Abstract] OR humanistic[Title/Abstract] OR qualitative[Title/Abstract])	
PsycNet	Title: anhedonia AND Abstract: depressive disorder OR Abstract: MDD OR Abstract: depression AND Abstract: quality of life OR Abstract: QoL OR Abstract: HRQoL OR Abstract: function* OR Abstract: impact* OR Abstract: well being OR Abstract: general health perception* OR Abstract: health state* OR Abstract: self report* OR Abstract: patient report* OR Abstract: PRO OR Abstract: PROs OR Abstract: diary OR Abstract: diaries OR Abstract: patient perspective* OR Abstract: patient centricity OR Abstract: patient centered OR Abstract: patient centered OR Abstract: interview* OR Abstract: survey* OR Abstract: conceptual model* OR Abstract: disease model* OR Abstract: focus group* OR Abstract: literature review* OR Abstract: systematic review* OR Abstract: longitudinal OR Abstract: observational OR Abstract: cohort* OR Abstract: case series OR Abstract: humanistic OR Abstract: qualitative	
Patient blogs/forums	**Category**	Search string
	Time frame	Last 10 years
	Disease	Anhedonia AND (depressive disorder OR MDD OR depression)
	Outcomes	Signs OR symptoms OR impacts OR experience
ClinicalTrials.gov	Condition or disease	anhedonia
	Other terms	depressive disorder OR MDD OR depression
	Study type	All studies
	Age group	Adult (18–64 years)
PROQOLID and PROLABELS	anhedonia	

MDD: major depressive disorder; PRO: patient-reported outcome; QoL: quality of life

**Table 2:** Targeted literature review eligibility criteria

Inclusion criteria	Exclusion criteria
• MDD patients with anhedonia • Adult patients (18–64 years of age)	• Pediatric patients (<17 years of age) • Older adult patients (65 years of age) • Non-human study
• Disease- and/or treatment-related signs, symptoms, and/or impacts experienced by patients with anhedonia in MDD • Use of PROs of interest • Development and psychometric evaluation of PROs of interest	• Focus on pathogenesis, genetics, molecular biology, or biomarkers only
• Non-interventional/qualitative studies, including patient interview and/or focus group studies • Review studies • Observational studies, including registries and real-world data • Interventional studies, including clinical trials • Development and/or psychometric evaluation studies for PROs of interest	• NA

MDD: major depressive disorder; NA: not applicable; PRO: patient-reported outcome

Article review results were further supplemented with searches in online patient blogs and forums. Specifically, patient blogs and forums were searched for patient-created content about their experiences with anhedonia in the context of MDD. Blogs and forums associated with national patient organizations and posts within the last 10 years were prioritized.

Specific to PRO identification, the search strategy and inclusion/exclusion criteria were applied in ClinicalTrials.gov (further supplemented by TrialTrove), PROQOLID and PROLABELS via ePROVIDE (further supplemented by PharmaProjects), PubMed, and PyscNet, and results were reviewed for relevant content (i.e., PROs assessing anhedonia).

Identified patient-relevant concepts were used to develop a preliminary conceptual model of the patient experience with anhedonia in the context of MDD. The model was then used to conduct concept and item-mapping exercises with the identified PROs to assess concept coverage.

### Clinician interviews

Clinicians were identified through the TLR, IQVIA, and Neurocrine databases, and a recruitment vendor. Clinicians were recruited if they were conducting research and/or treating at least 10 patients with anhedonia per month. A 60-minute, one-on-one telephone interview with individual clinicians was conducted to understand their experience with patients reporting anhedonia in the context of MDD, including related signs, symptoms, and impacts. A semi-structured discussion guide was used during the interview and participating clinicians were compensated for their time.

Using the discussion guide, clinicians were asked to discuss their clinical background and experience with patients with anhedonia in the context of MDD, including relevant treatment options and how they assessed change with treatment. Clinicians were also asked about the signs, symptoms, and impacts of anhedonia in the context of MDD observed in or reported by their patients. Once all spontaneously reported concepts were exhausted, the preliminary conceptual model derived through the TLR was presented to clinicians and they were asked to comment on the included concepts.

### Participant interviews

#### Participant sample

Participants were recruited using a third-party recruitment vendor, Global Perspectives, through the vendor’s databases and clinician networks. The vendor shared a recruitment flyer that provided an overview of the study with potential participants. Using a screener form, potential participants were screened per the following eligibility criteria: consented to participate in this study and provided authorization to disclose health information; aged 18–65 years; current diagnosis of MDD according to the Diagnostic and Statistical Manual of Mental Disorders, Fifth Edition (DSM-5) [26]; had been treated with antidepressant medication(s); continued to have clinically significant anhedonia (i.e., SHAPS ≥ 30; the SHAPS was not administered as part of the screening process and the score was provided for clinician reference only); and physically able to participate in a one-on-one, 90-minute telephone interview (conducted in one sitting) in English using an internet-enabled computer or other device with screen-sharing/viewing capabilities.

Individuals were ineligible if they met any of the exclusion criteria: had a current psychiatric disorder other than MDD, such as personality disorder, schizophrenia, schizoaffective disorder, other psychotic disorder, bipolar or eating disorders, dementia, intellectual disability, and/or mental disorder due to a general medical condition (as defined by the DSM-5) or had been treated with electroconvulsive therapy during the 6 months before screening (comorbid anxiety disorders were not considered exclusionary psychiatric disorders for this study); history of illicit drug or alcohol use disorder during the year before screening, per the judgment of the treating healthcare provider; current serious risk of suicide, self-harm, or aggressive behavior, per the judgment of the treating healthcare provider; unable to adhere to the requirements of this study and/or had a history of poor compliance in research studies.

Individuals who were considered potentially eligible based on the initial screener form provided written informed consent. Subsequently, each potential participant’s clinician completed a confirmation of diagnosis (CoD) form. The first part of the CoD form was used to confirm the participant’s agreement to have their clinician share relevant medical data with IQVIA for the purpose of confirming their eligibility to participate in an interview. The remainder of the form was used by the clinician to confirm the participant’s eligibility, including a current MDD diagnosis and presence of anhedonia. Once confirmed as eligible, participants were scheduled for interviews.

The 90-minute interview was facilitated by a trained IQVIA moderator using a semi-structured concept elicitation (CE)/cognitive debriefing (CD) discussion guide. Before the start of the interview, participants consented to be audio-recorded. During CD, the DARS was presented to each participant via screenshare during the interview. Once the interview was completed, participants were provided with an honorarium (US$150) and the audio recordings were transcribed.

The interview approach followed was in line with recommended guidelines provided by the International Society for Pharmacoeconomics and Outcomes Research (ISPOR) Good Research Practices Task Force [27, 28]. In addition, the study, including all study materials (research protocol, informed consent form, screener form, eligibility checklist, discussion guide, recruitment flyer, email correspondence, ovation honoraria communication) underwent Western Copernicus Group Institutional Review Board review and approval before use with participants (IRB #1312058).

### Hybrid concept elicitation/cognitive debriefing discussion guide

The semi-structured discussion guide included CE and CD sections and was designed to explore the patient experience with anhedonia in the context of MDD, as well as to evaluate content validity of the DARS. The guide included introductory text, open-ended questions, and probing questions, which were used to follow up on points of interest during discussions.

The CE section was designed to understand the most relevant and important signs, symptoms, and impacts of anhedonia in the context of MDD from the patient perspective. Participants were asked to discuss their experience with MDD, including anhedonia, diagnosis, and treatment. This was followed by an open-ended discussion on their signs, symptoms, and impacts of anhedonia (including frequency, duration, and how the concept may have changed over time). After all spontaneously reported concepts were exhausted, participants were probed on any remaining concepts in the preliminary conceptual model, as derived through the TLR and clinician interviews. Participants were asked to provide a disturbance rating on a scale from 0 = (sign/symptom/impact) does not disturb your life at all to 10 = (sign/symptom/impact) greatly disturbs your life for each reported concept and to identify their most bothersome signs/symptoms and impacts.

This was followed by the CD section, which was designed to assess participant understanding and use of the DARS content. The CD section was conducted using a think-aloud method, whereby the participants voiced their thought processes as they read and interpreted the content as well as provided responses to the items. As needed, the moderator asked follow-up questions on points of interest, including interpretation, relevance, and clarity of the instructions, items, and response options.

### Analysis

De-identified transcripts were generated from interview audio recordings. ATLAS.ti 8 Windows (ATLAS.ti Scientific Software Development GmbH) was used to code all transcripts. Participant-level data were categorized by codes and organized into an Excel spreadsheet for further analysis. Two coders participated in the coding process; all coded transcripts were then reviewed by the coding team lead to ensure coding consistency.

### Transcript coding

The transcripts were coded to identify participant-reported concepts, including the number of participants reporting each concept, mean disturbance ratings for concepts, and concepts reported as most bothersome. The transcripts were also coded to understand participant interpretation, clarity, and relevance of the instructions, items, and response options in the DARS.

The frequency of report per concept (total, spontaneous, and probed), mean disturbance rating, salience, and saturation were evaluated for all participant-reported concepts. Concepts were divided into two categories: signs/symptoms and impacts. Impacts were further categorized by domain. Concept salience was determined by mapping the number of participants who reported each concept against the mean disturbance rating for the concept. Concepts that were reported by ≥ 50.0% of participants and had a mean disturbance rating ≥ 5.0 (on a scale from 0 to 10) were considered salient (i.e., most relevant and important).

Saturation was defined as the point at which additional interviews in this population would not elicit any further novel concepts of the patient experience. To evaluate concept saturation, the 20 interviews were reviewed in four chronological waves of five participants each. The initial report of each concept was reviewed against each wave of interviews: the first wave (n = 5) was compared with the second wave (n = 5); these first and second waves were compared with the third wave (n = 5); the first, second, and third waves were then compared with the fourth wave (n = 5).

CD data for the DARS were categorized into three code groups: 1) interpretation codes that captured participant understanding of the content and whether it aligned with its intended meaning, 2) relevance codes that captured whether participants demonstrated that an item concept was relevant to their experience with anhedonia in the context of MDD, and 3) clarity codes that captured whether participants reported that the content was clear or unclear, and any suggested revisions if unclear. Relevance and clarity data were only considered if a participant interpreted the given content as intended. Any data that were not collected due to time constraints or data that were found to be inconclusive during the analyses were coded as “data not available”.

### Item mapping

The item-mapping exercise was designed to assess the concept coverage of the DARS against concepts included in the conceptual model. A preliminary item-mapping exercise was conducted during an interim analysis of the interview data, including input from the DARS developers (S.J.R. and S.H.K.), to review the conceptual model and to assess any potential gaps.

## Results

### Targeted literature review

In total, 129 articles were identified and 82 abstracts were selected for further review. Of these, 19 articles (Supplementary Table 1) met the eligibility criteria for full-text review and analysis. The full-text article review identified nine symptoms and six impacts of anhedonia in the context of MDD. The symptoms were organized by timeline category (i.e., before, during, and after the hedonic experience) and the impacts were organized by domain (i.e., activities of daily living, cognition, emotional, physical, social). In addition, patient blogs/posts were searched and five results were selected for review (two patient blogs, two patient vlogs, and one organization blog); two symptoms– *consummatory anhedonia* and *motivational anhedonia*– were identified in both the published literature and patient blogs/posts. All concepts identified via the full-text article review and patient blog/post review were subsequently used to develop the preliminary conceptual model.

A total of 97 clinical outcome assessments were identified, including 29 PROs with at least one anhedonia-related item, which were selected for further review. Of these 29 PROs, nine were identified as designed to assess anhedonia. Using the preliminary conceptual model, a gap analysis was conducted by mapping the nine PROs to the concepts included in the model. The DARS, Revised CSAS, and Revised CPAS were identified as the only three instruments that addressed a substantial portion of concepts (i.e., 42%, 37%, and 42%, respectively). However, the Revised CSAS and Revised CPAS were limited to social and physical anhedonia, respectively, and therefore were considered unsuitable for the context of use.

### Clinician interviews

In total, six clinicians who were treating patients or conducting research with patients with anhedonia in the context of MDD were interviewed. These clinicians reported that most of their patients (75.0%–95.0%) were treated with antidepressants and about 40.0%–60.0% of patients continued to experience significant anhedonia despite antidepressant treatment. Specific to anhedonia in the context of MDD, 13 symptoms and 25 impacts were endorsed by clinicians; of note, 10 symptoms and nine impacts were previously identified via the TLR. Clinicians identified 11 symptoms and impacts as the most bothersome to patients. When assessing meaningful change in anhedonia in the context of MDD, clinicians reported observing increased motivation, increased positivity, changes in daily life, and/or improvement in physical health. Two clinicians also described creating inventories of pleasurable activities specific to patients and looking for increased participation in the activities to assess meaningful change. The preliminary conceptual model was updated using the concepts endorsed by clinicians. All previously identified concepts (via the TLR) were retained, and novel concepts reported by clinicians were added to the model (Supplementary Fig. 1).

**Fig. 1:**
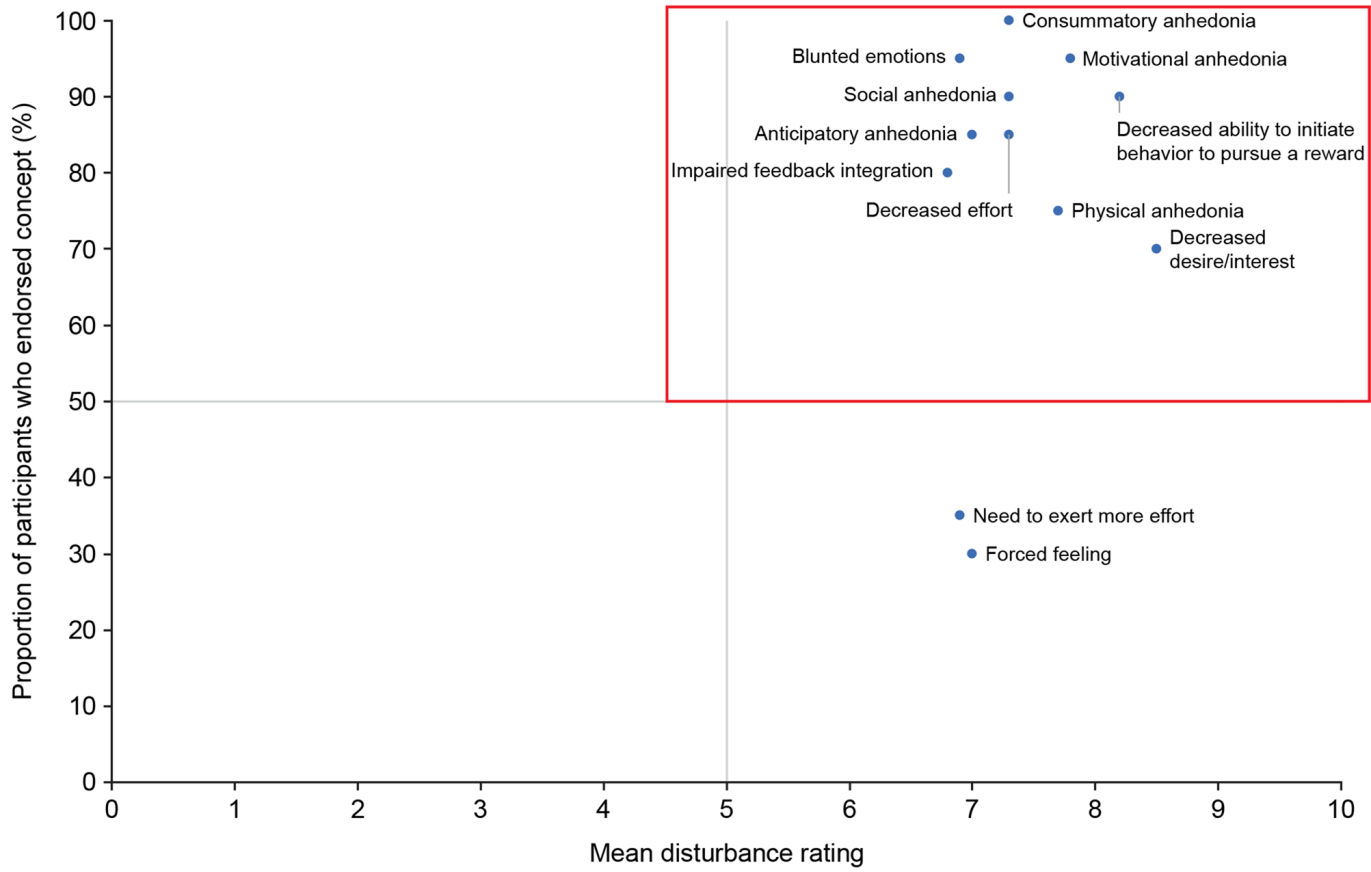
Mapping of participant report versus mean disturbance rating for symptoms

### Participant demographic and health information

Twenty participants completed an interview. The mean (standard deviation [SD]) age of participants was 48.5 (9.2) years. Participants had been diagnosed with MDD for a mean (SD) 16.9 (13.0) years prior. Over half of participants (n = 13, 65.0%) initiated antidepressant medication at the time of diagnosis; the remaining participants (n = 7, 35.0%) initiated antidepressant medication before or after diagnosis. Demographic and health information is detailed in Table 3.

**Table 3:** Demographic and health information for participants

Characteristic	Total N = 20 n (%)
**Age (years)**	
Mean (standard deviation)	48.5 (9.2)
Minimum–maximum	28–62
**Gender**	
Female	15 (75.0%)
Male	5 (25.0%)
**Race/ethnicity**	
White or Caucasian	12 (60.0%)
Hispanic or Latino/a	4 (20.0%)
Two or more races/ethnicities	3 (15.0%)
Black or African American	1 (5.0%)
**Work status** ^a^	
On disability	10 (50.0%)
Working part-time	6 (30.0%)
Working full-time	4 (20.0%)
Retired	3 (15.0%)
Student	2 (10.0%)
Seeking employment	1 (5.0%)
**Highest level of education achieved**	
Less than high school	1 (5.0%)
High school diploma	1 (5.0%)
Some college	7 (35.0%)
Associate degree	5 (25.0%)
Bachelor’s degree	4 (20.0%)
Master’s degree	1 (5.0%)
Doctorate degree	1 (5.0%)
**Living situation** ^a^	
Live alone	7 (35.0%)
Live with partner	7 (35.0%)
Live with roommates	4 (20.0%)
Live with family	3 (15.0%)
**Length of time since diagnosis (years)**	
Mean (standard deviation)	16.9 (13.0)
Minimum–maximum	<1–44 years
**Length of time between starting antidepressant medication and diagnosis (years)**	
Prior to diagnosis^b^	1 (5.0%)
At diagnosis	13 (65.0%)
<1 year after diagnosis	1 (5.0%)
1 year after diagnosis	1 (5.0%)
2–5 years after diagnosis	3 (15.0%)
10–15 years after diagnosis	1 (5.0%)

^a^Options were not mutually exclusive (i.e., some participants reported multiple work statuses/living situations)

^b^The participant formally received the diagnosis as an adult but began treatment as a minor (<18 years of age)

### CE data

#### Terminology

All participants (N = 20) reported using the term “depression” or “MDD”, and most (n = 16, 80.0%) were familiar with the term “anhedonia”.

#### Symptoms

Participants reported a total of 12 symptoms. Ten of the 12 symptoms were deemed salient (i.e., reported by ≥ 50% of participants and had a mean disturbance rating of ≥ 5.0), and are captured in the upper-right quadrant in Fig. 1. Further, three symptoms were highlighted as most bothersome by multiple participants: *physical anhedonia* (n = 6, 30.0%), *consummatory anhedonia* (n = 4, 20.0%), and *motivational anhedonia* (n = 3, 15.0%). The frequencies for all reported symptoms, including categorization of spontaneous versus probed frequency and whether highlighted as most bothersome, are summarized in Supplementary Table 2.

#### Impacts

**Fig. 2:**
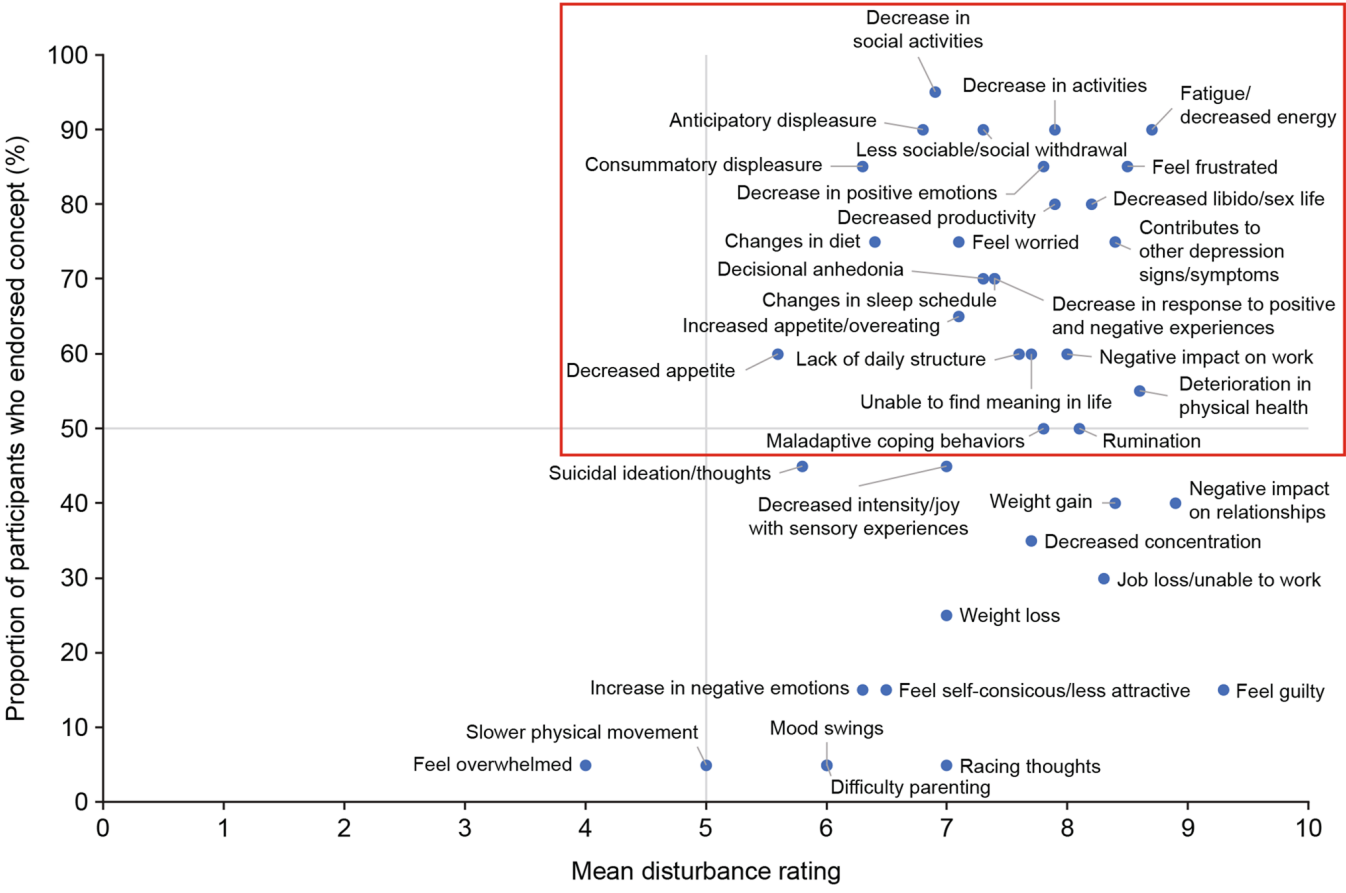
Mapping of participant report versus mean disturbance rating for impacts

Participants reported a total of 39 impacts, which were categorized into seven domains: activities of daily living, cognition, coping, emotional, physical, social, and work. Twenty-four impacts were deemed salient (i.e., reported by ≥ 50% of participants and had a mean disturbance rating of ≥ 5.0) and are captured in the upper-right quadrant of Fig. 2. Four impacts were highlighted as most bothersome by at least three participants each: *decreased social activities* (n = 7, 35.0%), *decreased libido/sex life* (n = 6, 30.0%), *changes in sleep schedule* (n = 3, 15.0%), *fatigue/decreased energy* (n = 3, 15.0%). Frequencies for all reported impacts, including categorization of spontaneous versus probed frequency and whether highlighted as most bothersome, are summarized in Supplementary Table 3.

#### Saturation of symptoms and impacts

All 12 symptoms were initially reported in the first wave of interviews and saturation was considered achieved for symptoms. Of the 39 impacts, 30 were initially reported in the first wave, followed by eight in the second wave. No novel impacts were reported in the third wave. One novel impact (*feel self-conscious/less attractive* [n = 3, 15.0%]) was reported in the fourth wave. Two participants (n = 2, 10.0%) described this concept as distal (*feeling self-conscious/less attractive* due to their *weight gain*) and not directly associated with anhedonia. With this, it was unlikely that proximal novel concepts would be reported through additional interviews and saturation was considered achieved for impacts.

#### Final conceptual model

**Fig. 3:**
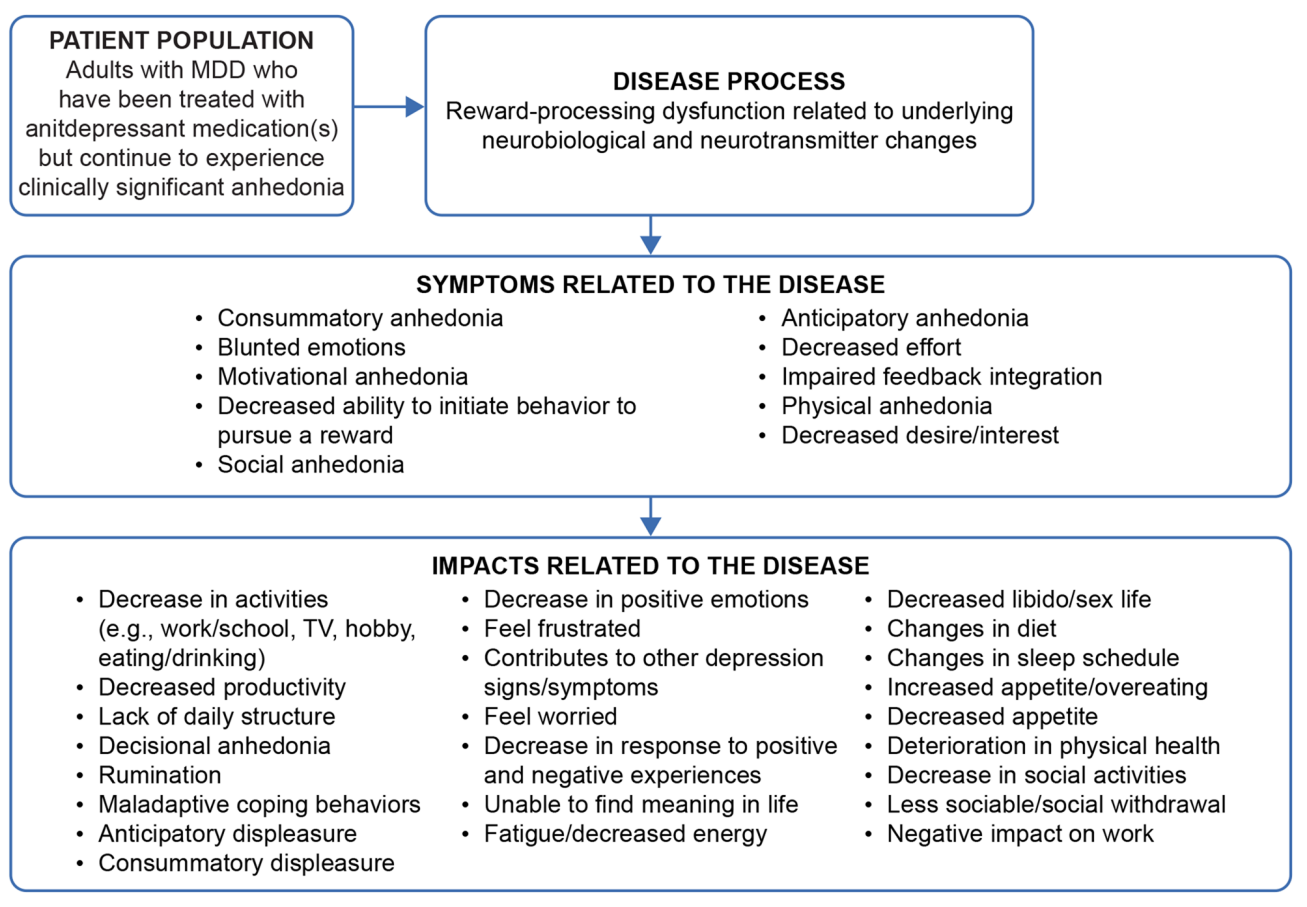
Final conceptual model updated through the participant interviews. MDD: major depressive disorder

The preliminary conceptual model developed through the TLR and clinician interviews was further refined and finalized through the participant interviews. All 34 salient concepts identified via the participant interviews were included in the final conceptual model (Fig. 3). Of these concepts, 18 were initially identified via the TLR and 15 were initially identified via the clinician interviews; the final concept was identified via the participant interviews. The iterative consistency in the identification of the 34 concepts across the research activities (i.e., TLR, clinician interviews, participant interviews) supports the robustness of the final conceptual model.

#### CD data

CD was conducted with all participants (N = 20). Due to time constraints, one of the 20 participants debriefed only DARS items 1–4; the remaining 19 participants debriefed all DARS items. Across item debriefing, data were not available for up to 5.0–10.0% of participants.

#### Instructions

All participants (100.0%) interpreted the instructions as intended. Additionally, most (75.0%) interpreted and used the recall period *right now* as intended. When discussing the recall period, the remaining participants (25.0%) also considered the previous week/month. Of note, these participants contextualized their definition of the recall period to demonstrate that it reflected their current “state”. Almost all participants (95.0%) reported that the instructions were clear; the remaining participant (5.0%) described that the instructions were unclear because they were unsure how to respond to items if they felt differently about the examples they provided in response to the domain prompts. Despite this initial feedback, the participant interpreted the instructions as intended and was able to respond to the items in the DARS without clarification from the moderator.

#### Items

All 17 items in the DARS were interpreted as intended by most participants (90.0–100.0%) and reported to be clear (78.9–100.0%). Similarly, all four domain instructions (i.e., instructing respondents to consider their favorite activities per domain) were interpreted as intended by all participants (100.0%) and found to be clear by most (89.5–100.0%). All response options (i.e., *Not at all, Slightly, Moderately, Mostly, Very much*) were interpreted as intended by most participants (95.0–100.0%) and found to be clear (95.0%). Item concepts were described as relevant by most participants (73.7–100.0%). See Table 4 for a summary of the CD results and Supplementary Table 4 for results per instructions and items.

**Table 4:** Summary of participant-reported content validity feedback for the DARS

DARS content	Characteristic	Percentage of Participants (%)
Instructions	Interpreted as intended	95.0–100.0
	Clear	95.0
Domain instructions	Interpreted as intended	100.0
	Clear	89.5–100.0
Items	Interpreted as intended	90.0–100.0
	Clear	78.9–100.0
	Relevant	73.7–100.0
Response options	Interpreted as intended	95.0–100.0
	Clear	95.0

DARS: Dimensional Anhedonia Rating Scale

While most of the DARS content (i.e., domain instructions, items, response options) was generally well understood and clear, some issues were demonstrated by participants. Notably, multiple specificity and/or relevance issues were identified for item 7 (*I want to have these foods/drinks*), item 8 (*I would eat as much of these foods/drinks as I could*), and item 10 (*I would be interested in doing things that involve other people*). When responding to item 7, four participants (21.1%) considered their health or other MDD symptoms in their response (e.g., “I’d say probably ‘mostly’ because I probably can’t have them every day because I know that it wouldn’t be healthy for me necessarily, or not all three in one day”). When responding to item 8, 11 participants (57.9%) considered or described confusion on whether to consider their health, other depression symptoms, and/or finances (e.g., “I do have some symptoms of disordered eating that relate to my depression… eating as much of a food as I could is usually not a good thing. It’s not related to me enjoying the food”). When responding to item 10, four participants (21.1%) considered or described confusion on whether to consider activities beyond the examples they provided in response to the social activities domain (e.g., “The two [examples] that I’m talking about or it could also be in generally”).

### General impressions of the DARS

Most participants (84.2%) reported that it was easy to provide at least two examples per domain in the DARS; one participant (5.3%) reported that it was neither easy nor difficult and two participants (10.5%) reported that it was difficult. The majority of participants (78.9%) found it easy to answer each question about the examples they provided in response to the domain prompts collectively, and three participants (15.8%) reported that it was difficult. However, all participants (100.0%) were able to complete the DARS successfully. Most participants (89.5%) reported that the DARS was comprehensive (i.e., not missing concepts related to their anhedonia).

### Mapping of the DARS to the conceptual model

Through the preliminary item mapping and related input received from the DARS developers, a novel concept in the interview data (i.e., *decreased desire/interest*) was identified. This concept was subsequently included in the analysis conducted with the complete interview sample.

With the complete interview sample (N = 20), the item mapping exercise was reconducted using the final conceptual model, which showed that the DARS provided concept coverage for eight of the 10 salient symptoms and for two of the 24 salient impacts included in the model. The two symptoms that were not assessed by the DARS were *blunted emotions* and *impaired feedback integration*. Participant descriptions of *blunted emotions* suggested that the concept applied to both positive and negative emotions and thus had overlap with *consummatory anhedonia*, which is covered by the DARS. Specific to *impaired feedback integration*, participants reported it only after probing and some participants described that they were not aware of this experience in real time; thus, it was not considered as a suitable concept for assessment by patients. Given the DARS was designed to assess symptoms of anhedonia, it was not anticipated that it would provide concept coverage for impacts.

## Discussion

The DARS is a 17-item PRO instrument designed to assess anhedonia. It has demonstrated ability to differentiate between healthy and depressed groups and psychometric analysis has demonstrated good internal consistency, convergent validity, and divergent validity; however, the DARS has not undergone analysis for test-retest reliability. Moreover, the DARS had not previously undergone content validity testing with patients [22].

This study aimed to characterize the patient experience with anhedonia in the context of MDD and to evaluate content validity of the DARS. The methods used for this study were in line with FDA and ISPOR guidance on identifying and evaluating PROs as content valid and fit for purpose [27–29]. Given the robust development and prior testing of the DARS, it was anticipated that it would perform well with patients in terms of content validity. With this, the results of this study aimed to further build upon the demonstrated measurement properties of the DARS.

The TLR, clinician interviews, and participant CE/CD interviews were conducted to better understand the patient experience with anhedonia in the context of MDD. The TLR was first used to identify signs, symptoms, and impacts of anhedonia in the context of MDD in published literature and online patient blogs/forums, and subsequently develop a preliminary conceptual model of the patient experience. The signs, symptoms, and impacts were further explored through the clinician interviews, and the preliminary conceptual model was updated.

Insights derived from the TLR and clinician interviews informed content development for the participant interviews, including the symptoms and impacts (as included in the preliminary conceptual model) for discussion with participants. In line with FDA and ISPOR guidance, participant interviews provide the most direct and comprehensive way to characterize the patient experience and are thus crucial to finalize disease conceptual models. Further, participant interviews are necessary to evaluate and establish content validity of PROs, such as the DARS.

Through the participant interviews, 12 symptoms and 39 impacts were identified. Saturation was considered achieved for both symptoms and impacts. Of the concepts reported, 10 symptoms and 24 impacts were deemed salient (i.e., reported by ≥ 50.0% of participants with a mean disturbance rating ≥ 5.0, suggesting that these concepts are most relevant and important to participants), and used to inform the final conceptual model. A total of 34 concepts were included in the model, encapsulating the patient experience with anhedonia in the context of MDD. Using the conceptual model, the item-mapping exercise conducted with the DARS demonstrated that the DARS provides suitable concept coverage (i.e., adequately assesses symptoms) in this patient population.

CD of the DARS demonstrated that the instructions, items, and response options were generally well understood, relevant, and clear to participants. Of note, some participants demonstrated issues related to specificity and/or relevance with items 7, 8, and 10, as they considered factors beyond their anhedonia or provided examples (i.e., examples they provided in response to the domain prompts) when responding to the items. The identified issues may confound the items’ ability to accurately assess change in anhedonia; thus, it is recommended that these items be further evaluated through planned psychometric analyses.

As a limitation of this study, the DARS was presented to participants via screensharing, and participants responded verbally. In a clinical trial setting, respondents would write or record (via pen and paper or an electronic device, respectively) their examples when completing the DARS. To mitigate this, the moderator recorded the examples on behalf of participants and reminded them of their provided examples as needed. Despite this distinction, all participants were able to successfully complete the DARS through screenshare and verbal discussion. Another limitation would be the potential for limited generalizability to individuals with MDD and comorbid mental/medical conditions, given that participants with comorbid mental/medical conditions were excluded from this study; further research may help support DARS usage across a broader population of individuals with MDD.

The current study demonstrates content validity of the DARS in adults with anhedonia in the context of MDD. Compared to the limitations of traditionally used PRO instruments designed to assess anhedonia [12, 20], the DARS has been shown to provide a more generalizable and applicable way to assess state anhedonia through patient self-reporting. This demonstration of content validity adds to the growing body of work supporting the measurement properties of the DARS. It also supports the DARS as a PRO instrument that can be used to assess anhedonia consistently and reliably in clinical trial settings and ultimately support the evaluation of treatment efficacy.

## Conclusions

- A conceptual model of the patient experience with anhedonia in the context of MDD was developed.
- Content validity of the DARS was established.
- The DARS provides suitable concept coverage for the context of use.

## Electronic supplementary material

Below is the link to the electronic supplementary material.

Supplementary Material 1

## Data Availability

The dataset(s) supporting the conclusions of this article is(are) included within the article (and its additional file(s)).

## Abbreviations

CD: Cognitive debriefing
CE: Concept elicitation
CoD: Confirmation of diagnosis
CPAS: Chapman Physical Anhedonia Scale
CSAS: Chapman Social Anhedonia Scale
DARS: Dimensional Anhedonia Rating Scale
DSM-5: Diagnostic and Statistical Manual of Mental Disorders, Fifth Edition
FDA: Food and Drug Administration
ISPOR: International Society for Pharmacoeconomics and Outcomes Research
PRO: Patient-reported outcome
SHAPS: Snaith-Hamilton Pleasure Scale
TLR: Targeted literature review
