# Supplementary material for: Establishing content validity of the Dimensional Anhedonia Rating Scale
Source: J Patient Rep Outcomes. 2025 Mar 11;9:31. doi: 10.1186/s41687-025-00860-x (PMC11896914; doi:10.1186/s41687-025-00860-x)
Supplement: Supplementary file 1 — Supplementary Material 1 [file 41687_2025_860_MOESM1_ESM.docx]

**Supplementary material**

**Establishing content validity of the Dimensional Anhedonia Rating Scale**
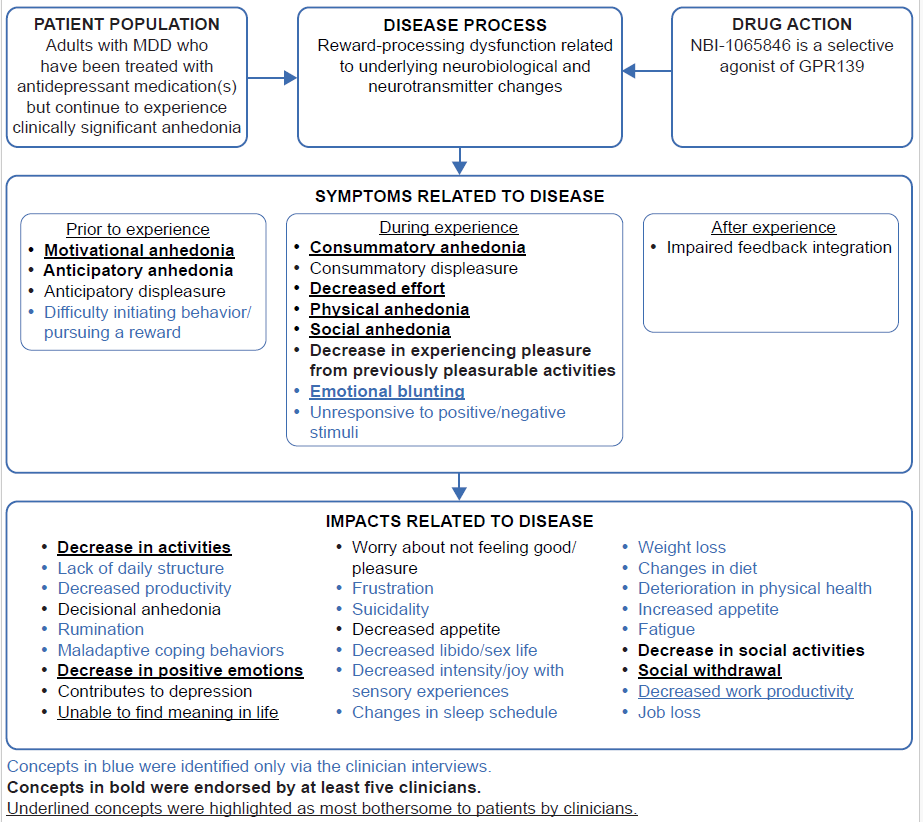


Supplementary Fig. 1 Preliminary conceptual model developed through the targeted literature review and clinician interviews

MDD: major depressive disorder.

**[Supplementary Table 1](#supplementary_table_3)** [Full-text article review reference list](#supplementary_table_3)

| 1. Argyropoulos SV, Nutt DJ (2013) Anhedonia revisited: is there a role for dopamine-targeting drugs for depression? J Psychopharmacol 27:869–877. doi: 10.1177/0269881113494104. Epub 2013 Jul 31. PMID: 23904408. |
| --- |
| 1. Chentsova-Dutton YE, Choi E, Ryder AG, Reyes J (2015) "I felt sad and did not enjoy life": cultural context and the associations between anhedonia, depressed mood, and momentary emotions. Transcult Psychiatry 52:616-635. doi: 10.1177/1363461514565850. Epub 2015 Jan 20. PMID: 25603917. |
| 1. Fehnel SE, Forsyth BH, DiBenedetti DB, Danchenko N, François C, Brevig T (2016) Patient-centered assessment of cognitive symptoms of depression. CNS Spectr 21:43-52. doi: 10.1017/S1092852913000643. Epub 2013 Sep 25. PMID: 24067243. |
| 1. Gao K, Sweet J, Su M, Calabrese JR (2017) Depression severity and quality of life of qualified and unqualified patients with a mood disorder for a research study targeting anhedonia in a clinical sample. Asian J Psychiatr 27:40-47. doi: 10.1016/j.ajp.2017.02.013. Epub 2017 Feb 14. PMID: 28558894. |
| 1. Heininga VE, Dejonckheere E, Houben M, Obbels J, Sienaert P, Leroy B, van Roy J, Kuppens P (2019) The dynamical signature of anhedonia in major depressive disorder: positive emotion dynamics, reactivity, and recovery. BMC Psychiatry 19:59. doi: 10.1186/s12888-018-1983-5. PMID: 30736751; PMCID: PMC6368777. |
| 1. Heininga VE, van Roekel E, Ahles JJ, Oldehinkel AJ, Mezulis AH (2017) Positive affective functioning in anhedonic individuals' daily life: Anything but flat and blunted. J Affect Disord 218:437-445. doi: 10.1016/j.jad.2017.04.029. Epub 2017 Apr 21. PMID: 28531841. |
| 1. Ho N, Sommers M. Anhedonia: a concept analysis (2013) Arch Psychiatr Nurs 27:121-9. doi: 10.1016/j.apnu.2013.02.001. Epub 2013 Apr 24. PMID: 23706888; PMCID: PMC3664836. |
| 1. Lambert C, Da Silva S, Ceniti AK, Rizvi SJ, Foussias G, Kennedy SH (2018) Anhedonia in depression and schizophrenia: a transdiagnostic challenge. CNS Neurosci Ther 24:615-623. doi: 10.1111/cns.12854. Epub 2018 Apr 23. PMID: 29687627; PMCID: PMC6489811. |
| 1. Lasch KE, Hassan M, Endicott J, Piault-Luis EC, Locklear J, Fitz-Randolph M, Pathak S, Hwang S, Jernigan K (2012) Development and content validity of a patient reported outcomes measure to assess symptoms of major depressive disorder. BMC Psychiatry 12:34. doi: 10.1186/1471-244X-12-34. PMID: 22534028; PMCID: PMC3386015. |
| 1. Liao A, Walker R, Carmody TJ, Cooper C, Shaw MA, Grannemann BD, Adams P, Bruder GE, McInnis MG, Webb CA, Dillon DG, Pizzagalli DA, Phillips ML, Kurian BT, Fava M, Parsey RV, McGrath PJ, Weissman MM, Trivedi MH (2019) Anxiety and anhedonia in depression: Associations with neuroticism and cognitive control. J Affect Disord 245:1070-1078. doi: 10.1016/j.jad.2018.11.072. Epub 2018 Nov 14. PMID: 30699849. |
| 1. McCarrier KP, Deal LS, Abraham L, Blum SI, Bush EN, Martin ML, Thase ME, Coons SJ; PRO Consortium’s Depression Working Group (2016) Patient-centered research to support the development of the Symptoms of Major Depressive Disorder Scale (SMDDS): initial qualitative research. Patient 9:117-134. doi: 10.1007/s40271-015-0132-1. PMID: 26113249. |
| 1. McIntyre RS, Woldeyohannes HO, Soczynska JK, Maruschak NA, Wium-Andersen IK, Vinberg M, Cha DS, Lee Y, Xiao HX, Gallaugher LA, Dale RM, Alsuwaidan MT, Mansur RB, Muzina DJ, Carvalho AF, Jerrell JM, Kennedy SH (2016) Anhedonia and cognitive function in adults with MDD: results from the International Mood Disorders Collaborative Project. CNS Spectr 21:362-366. doi: 10.1017/S1092852915000747. Epub 2015 Dec 30. PMID: 26714651. |
| 1. Rizvi SJ, Pizzagalli DA, Sproule BA, Kennedy SH. Assessing anhedonia in depression: potentials and pitfalls (2016) Neurosci Biobehav Rev 65:21-35. doi: 10.1016/j.neubiorev.2016.03.004. Epub 2016 Mar 6. PMID: 26959336; PMCID: PMC4856554. |
| 1. Rømer Thomsen K, Whybrow PC, Kringelbach ML (2015) Reconceptualizing anhedonia: novel perspectives on balancing the pleasure networks in the human brain. Front Behav Neurosci 9:49. doi: 10.3389/fnbeh.2015.00049. PMID: 25814941; PMCID: PMC4356228. |
| 1. Sherdell L, Waugh CE, Gotlib IH (2012) Anticipatory pleasure predicts motivation for reward in major depression. J Abnorm Psychol 121:51-60. doi: 10.1037/a0024945. Epub 2011 Aug 15. PMID: 21842963; PMCID: PMC3335300. |
| 1. Treadway MT, Zald DH (2011) Reconsidering anhedonia in depression: lessons from translational neuroscience. Neurosci Biobehav Rev 35:537-555. doi: 10.1016/j.neubiorev.2010.06.006. Epub 2010 Jul 11. PMID: 20603146; PMCID: PMC3005986. |
| 1. Winer ES, Jordan DG, Collins AC (2019) Conceptualizing anhedonias and implications for depression treatments. Psychol Res Behav Manag 12:325-335. doi: 10.2147/PRBM.S159260. PMID: 31191054; PMCID: PMC6521843. |
| 1. Winer ES, Bryant J, Bartoszek G, Rojas E, Nadorff MR, Kilgore J (2017) Mapping the relationship between anxiety, anhedonia, and depression. J Affect Disord 221:289-296. doi: 10.1016/j.jad.2017.06.006. Epub 2017 Jun 13. PMID: 28668590; PMCID: PMC6080718. |
| 1. Wu H, Mata J, Furman DJ, Whitmer AJ, Gotlib IH, Thompson RJ (2017) Anticipatory and consummatory pleasure and displeasure in major depressive disorder: an experience sampling study. J Abnorm Psychol 126:149-159. doi: 10.1037/abn0000244. Epub 2016 Dec 12. PMID: 27936838; PMCID: PMC5305427. |

**Supplementary Table 2** Summary of participant-reported symptoms

| Concept | Total N=20  n (%) | Mean disturbance rating |
| --- | --- | --- |
| Consummatory anhedonia | 20 (100.0%)  *S=12, P=8* | 7.3^a^ |
| Blunted emotions | 19 (95.0%)  *S=4, P=15* | 6.9^a^ |
| Motivational anhedonia | 19 (95.0%)  *S=12, P=7* | 7.8^a^ |
| Decreased ability to initiate behavior to pursue a reward | 18 (90.0%)  *S=7, P=11* | 8.2^a^ |
| Social anhedonia | 18 (90.0%)  *S=14, P=4* | 7.3^a^ |
| Anticipatory anhedonia | 17 (85.0%)  *S=6, P=11* | 7.0^a^ |
| Decreased effort | 17 (85.0%)  *S=3, P=14* | 7.3^a^ |
| Impaired feedback integration | 16 (80.0%)  *S=0, P=16* | 6.8 |
| Physical anhedonia | 15 (75.0%)  *S=7, P=8* | 7.7^a^ |
| Decreased desire/interest | 14 (70.0%)  *S=9, P=5* | 8.5^a^ |
| Need to exert more effort | 7 (35.0%)  *S=7, P=0* | 6.9 |
| Forced feeling | 6 (30.0%)  *S=6, P=0* | 7.0^a^ |

^a^Indicates that at least one participant-reported this concept as most bothersome.
P: probed; S: spontaneous.

**Supplementary Table 3** Summary of participant-reported impacts

| Domain | Concept | Total N=20  n (%) | Mean disturbance rating |
| --- | --- | --- | --- |
| Activities of daily living | Decrease in activities (e.g., work/school, TV, hobby, eating/drinking) | 18 (90.0%)  *S=14, P=4** | 7.9 |
|  | Decreased productivity | 16 (80.0%)  *S=3, P=13* | 7.9^a^ |
|  | Lack of daily structure | 12 (60.0%)  *S=4, P=8* | 7.6 |
|  | Difficulty parenting | 1 (5.0%)  *S=1, P=0* | 6.0 |
| Cognition | Decisional anhedonia | 14 (70.0%)  *S=0, P=14* | 7.3 |
|  | Rumination | 10 (50.0%)  *S=4, P=6* | 8.1 |
|  | Decreased concentration | 7 (35.0%)  *S=7, P=0* | 7.7^a^ |
|  | Racing thoughts | 1 (5.0%)  *S=1, P=0* | 7.0 |
| Coping | Maladaptive coping behaviors | 10 (50.0%)  *S=3, P=7* | 7.8 |
| Emotional | Anticipatory displeasure | 18 (90.0%)  *S=2, P=16* | 6.8^a^ |
|  | Consummatory displeasure | 17 (85.0%)  *S=2, P=15* | 6.3 |
|  | Decrease in positive emotions | 17 (85.0%)  *S=6, P=11* | 7.8^a^ |
|  | Feel frustrated | 17 (85.0%)  *S=7, P=10* | 8.5^a^ |
|  | Contributes to other depression signs/symptoms | 15 (75.0%)  *S=1, P=14* | 8.4 |
|  | Feel worried | 15 (75.0%)  *S=3, P=12* | 7.1^a^ |
|  | Decrease in response to positive and negative experiences | 14 (70.0%)  *S=1, P=13* | 7.4^a^ |
|  | Unable to find meaning in life | 12 (60.0%)  *S=4, P=8* | 7.7^a^ |
|  | Suicidal ideation/thoughts | 9 (45.0%)  *S=2, P=7* | 5.8 |
|  | Feel guilty | 3 (15.0%)  *S=3, P=0* | 9.3 |
|  | Feel self-conscious/less attractive | 3 (15.0%)  *S=3, P=0* | 6.5 |
|  | Increase in negative emotions | 3 (15.0%)  *S=3, P=0* | 6.3 |
|  | Feel overwhelmed | 1 (5.0%)  *S=1, P=0* | 4.0 |
|  | Mood swings | 1 (5.0%)  *S=1, P=0* | 6.0 |
| Physical | Fatigue/decreased energy | 18 (90.0%)  *S=13, P=5* | 8.7^a^ |
|  | Decreased libido/sex life | 16 (80.0%)  *S=7, P=9* | 8.2^a^ |
|  | Changes in diet | 15 (75.0%)  *S=6, P=9* | 6.4 |
|  | Changes in sleep schedule | 14 (70.0%)  *S=3, P=11* | 7.4^a^ |
|  | Increased appetite/overeating | 13 (65.0%)  *S=6, P=7* | 7.1 |
|  | Decreased appetite | 12 (60.0%)  *S=8, P=4* | 5.6^a^ |
|  | Deterioration in physical health | 11 (55.0%)  *S=1, P=10* | 8.6^a^ |
|  | Decreased intensity/joy with sensory experiences | 9 (45.0%)  *S=0, P=9* | 7.0 |
|  | Weight gain | 8 (40.0%)  *S=7, P=1* | 8.4^a^ |
|  | Weight loss | 5 (25.0%)  *S=1, P=4* | 7.0^a^ |
|  | Slower physical movement | 1 (5.0%)  *S=1, P=0* | 5.0 |
| Social | Decrease in social activities | 19 (95.0%)  *S=16, P=3* | 6.9^a^ |
|  | Less sociable/social withdrawal | 18 (90.0%)  *S=16, P=2* | 7.3^a^ |
|  | Negative impact on relationships | 8 (40.0%)  *S=7, P=1* | 8.9^a^ |
| Work | Negative impact on work | 12 (60.0%)  *S=4, P=8* | 8.0^a^ |
|  | Job loss/unable to work | 6 (30.0%)  *S=2, P=4* | 8.3^a^ |

^a^Indicates that at least one participant-reported this concept as most bothersome.
P: probed; S: spontaneous.

**Supplementary Table T4** Summary of participant-reported content validity feedback per DARS domain instructions and items

| **Domain instructions and items** | **Interpreted as intended,**  **n (%)** | **Reported as clear, n (%)** | **Demonstrated as relevant, n (%)** |
| --- | --- | --- | --- |
| **Domain A – Please list at least 2 of your favorite pastimes/hobbies that are NOT primarily social** | **20 (100.0)^a^** | **20 (100.0)^a^** | **NA** |
| Item 1 – *I would enjoy these activities* | 19 (95.0) | 19 (95.0) | 17 (85.0) |
| Item 2 – *I would spend time doing these activities* | 18 (90.0) | 17 (85.0) | 18 (90.0) |
| Item 3 – *I want to do these activities* | 20 (100.0) | 20 (100.0) | 17 (85.0) |
| Item 4 – *These activities would interest me* | 19 (95.0) | 19 (95.0) | 15 (75.0) |
| **Domain B – Please list at least 2 of your favorite foods/drinks** | **19 (100.0)** | **17 (89.5)** | **NA** |
| Item 5 – *I would make an effort to get/make these foods/drinks* | 19 (100.0) | 18 (94.7) | 16 (84.2) |
| Item 6 – *I would enjoy these foods/drinks* | 18 (94.7) | 17 (89.5) | 14 (73.7) |
| Item 7 – *I want to have these foods/drinks* | 19 (100.0) | 18 (94.7) | 18 (94.7) |
| Item 8 – *I would eat as much of these foods/drinks as I could* | 19 (100.0) | 18 (94.7) | 17 (89.5) |
| **Domain C – Please list at least 2 of your favorite social activities** | **19 (100.0)** | **19 (100.0)** | **NA** |
| Item 9 – *Spending time doing these things would make me happy* | 19 (100.0) | 19 (100.0) | 19 (100.0) |
| Item 10 – *I would be interested in doing things that involve other people* | 19 (100.0) | 18 (94.7) | 19 (100.0) |
| Item 11 – *I would be the one to plan these activities* | 19 (100.0) | 18 (94.7) | 19 (100.0) |
| Item 12 – *I would actively participate in these social activities* | 19 (100.0) | 19 (100.0) | 19 (100.0) |
| **Domain D – Please list at least 2 of your favorite sensory experiences** | **19 (100.0)** | **19 (100.0)** | **NA** |
| Item 13 – *I would actively seek out these experiences* | 18 (94.7) | 17 (89.5) | 17 (89.5) |
| Item 14 – *I get excited thinking about these experiences* | 19 (100.0) | 18 (94.7) | 18 (94.7) |
| Item 15 – *If I were to have these experiences I would savor every moment* | 19 (100.0) | 15 (78.9) | 15 (78.9) |
| Item 16 – *I want to have these experiences* | 19 (100.0) | 18 (94.7) | 17 (89.5) |
| Item 17 – *I would make an effort to spend time having these experiences* | 18 (94.7) | 17 (89.5) | 17 (89.5) |

Data presented for Domain A and items 1–4 reflect the data collected from 20 participants. Data presented in Domains B, C, and D and items 5–17 reflect the data collected from 19 participants, as one participant did not debrief these sections and items due to time constraints.

^a^Interpretation and clarity data specific to the domain instructions.
DARS: Dimensional Anhedonia Rating Scale; NA: not applicable.
